# Features of successful bids for funding of applied health research: a cohort study

**DOI:** 10.1186/1478-4505-12-54

**Published:** 2014-09-22

**Authors:** Sheila Turner, Peter Davidson, Louise Stanton, Victoria Cawdeary

**Affiliations:** Wessex Institute, University of Southampton, Alpha House, Enterprise Road, Southampton, SO16 7NS UK; University of Southampton Clinical Trials Unit, Southampton, SO16 6YD UK

**Keywords:** Funding applications, Health technology assessment, Primary research, Research funding

## Abstract

**Background:**

The literature suggests that research funding decisions may be influenced by criteria such as gender or institution of the principal investigator (PI). The aim of this study was to investigate the association between characteristics of funding applications and success when considered by a research funding board.

**Methods:**

We selected a retrospective cohort of 296 outline applications for primary research (mainly pragmatic clinical trials) submitted to the commissioning board of the National Institute for Health Research (NIHR) Health Technology Assessment (HTA) Programme between January 1^st^ 2006 and December 31^st^ 2009. We selected proposals submitted to the commissioned NIHR HTA work stream as they addressed issues which the programme already deemed to be important, hence the priority of the research question was not considered as one of the selection criteria for success or failure. Main outcome measures were success or failure at short-listing and in obtaining research funding.

**Results:**

The characteristics of applications associated with success at shortlisting and funding were multi-disciplinarity of the team (OR 19.94 [5.13, 77.50], *P* <0.0001), particularly inclusion of a statistician (OR 3.76 [2.21, 6.37], *P* <0.0001), and the completion of a pilot/feasibility study (OR 4.11 [1.24, 13.62], *P* = 0.0209). The gender of the PI was not associated with success or failure at either stage. The PI’s affiliation institution was not associated with success or failure at shortlisting.

**Conclusions:**

The gender of the PI was not associated with success or failure. The characteristics of research applications most strongly associated with success were related to the range of expertise in the team and the completion of a pilot or feasibility study.

## Background

For most researchers, the task of obtaining funding for research is a vital and constant part of their working lives. The importance of writing a good application and selecting an appropriate funding body [1–6] has long been understood. Some of the issues relating to obtaining research funding, such as the aims of funding organisations [7–10], their policies [11, 12], priorities [10, 13, 14], processes [10, 15], and value for money [16–19], have previously been examined.

A system of peer review and selection by boards or committees is common to many funding organisations internationally [20–22]. It is in the interests of the funding organisations, researchers, and society in general that there is a transparent and fair way of selecting the best applications from those received. However, it is not always clear if the system is operating in the way intended, or if there may be unintentional outcomes in the system [23].

Previous analysis conducted over the last 10 to 12 years (mainly in the USA), suggests that in medical and biomedical research criteria, such as gender [24–27] or institution [28] of the principal investigator (PI) and the amount of funding available [29], may have an influence on success or failure in obtaining funding. Some previous studies of medical research have found a gender disparity in terms of the numbers of applications received from male and female PIs [24, 26, 27, 30], the amounts awarded [24, 25], and the success rate [24]. Success rate was related to seniority [24, 26, 30, 31] and qualifications [25, 32] of the applicants, which were both associated with gender [24, 25, 27, 33, 34]. However, a large meta-analysis considering gender differences in peer review of grant applications across a range of disciplines [35], and a later study [36], found no gender differences.

In view of the uncertainty about the current preferences of funding committees, this study aims to investigate the association between characteristics of funding applications and success in obtaining funding from one research funding organisation, the National Institute for Health research (NIHR) Health Technology Assessment (HTA) Programme, which was established in the UK in 1993. The NIHR HTA Programme funds a large number of late phase clinical trials and complex evidence synthesis studies investigating the clinical and cost effectiveness of a diverse range of interventions which may include drugs, devices, physical therapies, talking therapies, preventative interventions, surgical procedures, and tests. Because the NIHR embraces the principles of Athena Swan [37], we wished to investigate whether there was any disparity with regard to gender. From an informal examination of UK research council websites, the host institutions of their applicants and those applying to the NIHR HTA programme are broadly similar. We therefore anticipate that the results of this study will be useful to researchers preparing applications to this and similar funders and to research funders who may wish to improve funding systems.

## Methods

We undertook a retrospective cohort study of funding applications submitted to the NIHR HTA Commissioning Board, a committee of just over 20 independent senior academic clinicians and methodologists, selected for their experience in carrying out high quality health research. The board considers only commissioned research applications in which the research question is decided by the funding organisation, which advertises for applications for studies to answer the detailed research question specified. This advertisement includes a commissioning brief that gives details relating to the research question, participants, intervention, comparator, and outcome measures, as well as the suggested study type. The remit of the NIHR HTA Programme is described on the website [38]. This funding mode is in contrast to response-mode funding where researchers are free to submit applications on topics chosen by them. We used commissioned research so that we could examine generic factors in the teams and their applications, without having to take account of the importance of the topic they were proposing to investigate, as the topic had already been deemed important by the funder. Applications in response to a particular commissioning brief were in direct competition with each other, and funding decisions made on the quality of the application alone, as other variables such as research question and study outline framework had been indicated by the brief.

### Funding board processes

The funding process for the NIHR HTA programme comprises two stages. At the first stage, outline applications are considered by the board and the most promising are selected (or shortlisted) to submit a full application for further review by the board before a decision on which, if any, of the applications submitted should be funded. Comments on the quality of the application including adherence to the brief are formally noted. At the second stage, funding decisions are made after external peer review and subsequent consideration of the full applications by the board. At the outline stage, it is usual practice for the board to offer advice which is fed back to shortlisted applicants in order to improve the quality of the subsequent full application in light of these comments.

### Eligibility and sample size

**Figure 1:**
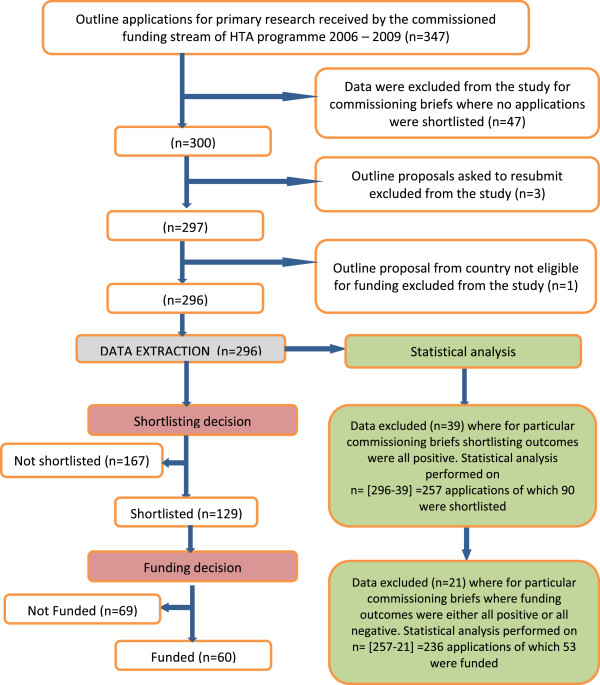
**Flowchart showing flow of applications through application and funding process and numbers of applications included in statistical analysis.**

Eligible applications were outline applications for primary research submitted to the Commissioning Board of the NIHR HTA programme from January 1^st^ 2006 to December 31^st^ 2009. Where no applications were shortlisted for a particular commissioning brief all data relating to that brief were excluded from the study. We also excluded from the study any outline proposals which the funding board asked to be resubmitted, and any applications received from countries not eligible for funding (Figure 1).

Only outline applications were included in this study as we wished to examine the applications as they were originally submitted by the researchers, before input from the board, to gain insight into which characteristics are predictive of success.

We selected the sample size to provide at least 10 events (in our case 10 applications funded) for each of 8 characteristics selected [39] (Table 1). Prior to starting the study we noted that there were 347 applications received by the NIHR HTA programme between January 1^st^ 2006 and December 31^st^ 2009 providing an adequate number of events. The study was not powered for the exploratory analysis of secondary characteristics, though these were adjusted for in the primary analysis.

**Table 1:** **Characteristics recorded**

	Characteristics on which study was powered
1	Gender of PI
2	Institution for PI
3	Number of co-applicants on application
4	Named statistician on application as a PI or co-applicant
5	Named clinician on application as a PI or co-applicant
6	Multi-disciplinarity of team – 2 or more out of:
	i) Health economist on application as a PI or co-applicant,
	ii) Clinician with NHS responsibilities on application as a PI or co-applicant
	iii) Social Scientist or Psychologist on application as a PI or co-applicant,
	iv) Methodologist on application as a PI or co-applicant
7	Has there been a pilot/feasibility study?
8	Has the PI or team led or participated in a relevant systematic review?

### Selection of characteristics

We conducted a literature search which identified research which suggested that criteria such as gender or the institution for the PI or skill mix for the team may have an influence on funding decisions. Medline, Health Management Information Consortium database, and CAB abstracts were searched via OVID (limits: English language, humans, 1996 onwards; search terms: funding, research grant, proposal, research bid, clinical trial, success; date of last search February 2012). The findings from the literature search were discussed by an external expert advisory group (please see acknowledgements). The characteristics of the applications to be recorded were selected *a priori* after this consultation process. Characteristics were selected in order to test hypotheses; we hoped that some of the characteristics, such as gender and institution for the first applicant would not influence outcome, whereas characteristics which might be expected to be associated with success included the composition of the team and whether or not a pilot or feasibility study had been completed. For a list of characteristics selected see Table 1.

In the UK, universities from the Russell Group ‘*which are committed to maintaining the very best research*’ [40], have received a high proportion of funding from research councils. In 2008/2009, Russell Group universities accounted for 68% (over £1.0 billion) of total income from the Research Councils [40]. Of the 20 (at the time of this cohort) Russell Group universities, six (known informally as the big six [THE 9/12/2010 http://www.timeshighereducation.co.uk/418215.article]) have been particularly successful in obtaining competitive funding. We examined whether having a first applicant from these groups was associated with application success.

We also selected other characteristics for adjustment in our model. These included clinical trials unit (CTU) involvement, total research costs and whether the study was multi-centred (which could be related to larger teams), history of NIHR HTA funding or NIHR HTA board members included on the application, both markers of expertise and experience, and whether the application met the brief and whether the form was filled in badly.

### Data extraction for selected characteristics

We extracted data relating to the selected characteristics (Table 1) of outline applications for primary research from paper files and recorded them in an MS Access database. A chart showing the flow of applications through the funding process is shown in Figure 1. We recorded data for most fields as yes or no and some as yes, no, or unclear. We categorised institution for the first applicant into three groups by research intensity: i) the big six (Universities of Cambridge, Edinburgh, Oxford, Manchester, Imperial College London, and University College London); ii) Russell group other than the big six (Universities of Birmingham, Bristol, Cardiff, Glasgow, Leeds, Liverpool, Newcastle, Nottingham, Sheffield, Southampton, Warwick, Kings College London, London School of Economics, and Queen’s Belfast); and iii) other universities.

We inferred gender of first applicant from their full name and title. Information as to whether or not the first applicant had previous NIHR HTA funding and whether any of the applicants were current or past board members at the time of submitting the application, was available from programme electronic records. We assessed adherence to the advertised commissioning brief from the written reports of the board. Whether the form was filled in badly was a judgement made considering numbers of spelling mistakes and typographical errors and how difficult it was to read the information presented. We categorised research cost into £500,000 bands.

We recorded two outcomes of interest: i) success at first stage of the application process where an outline proposal is assessed (short-listing) and ii) eventual success in obtaining funding, the final stage, in which a full proposal is seen by the board. Data regarding the first, success at the short-listing stage, was recorded in paper files; for the second, success in obtaining funding, we obtained data from electronic records.

One researcher (ST) initially performed the data extraction. Subsequently 30 (10%) of the applications were selected randomly and checked for accuracy by a second researcher (ES). Where it was unclear whether an application fulfilled a particular characteristic, differences were discussed by the team and decisions reached collectively. Of the 30 applications randomly selected and checked for accuracy, no discrepancies were found in 20 of the 24 fields (which included fields for factors for adjustment). A total of 8 discrepancies were found within only 4 fields [whether the team had lead or participated in a systematic review (2), methodologist (2), history of NIHR HTA grants (1), and CTU involvement (3)]. As the number of discrepancies was so low no further checks were made. The discrepancies were limited to those features that were open to interpretation, for instance, the disciplines of the applicants were sometimes not clear from job titles as an indication of specialism. We discussed uncertainties in these fields on a case by case basis and decisions were reached collectively within the research team. In the majority of cases where data extraction on the prior conduct of a pilot study or systematic review by the applicants was attempted, data were missing, so we recorded the characteristic as unclear.

Whether the form was filled in badly was a subjective judgement, however, as the data extraction was initially done by one researcher there was consistency within the sample. We only considered an application as badly completed if it contained multiple typographical errors or was laid out so poorly as to undermine confidence in the authors.

### Univariate and multivariate statistical analysis

We performed all analyses in STATA version 11.0. For the outcome “success at shortlisting” we excluded applications from the analysis if for a particular commissioning brief all the applications were shortlisted because they contributed nothing to knowledge about why one application for a brief was selected over another (Figure 1). For the same reason, for outcome “success obtaining funding” we excluded proposals from the analysis if either all the applications for a commissioning brief were funded or all were not funded (Figure 1). We fitted several regression models [39, 41–43] to test whether the selected characteristics were associated with an application being shortlisted or funded.

To do this, we used conditional logistic regression modelling instead of ordinary logistic regression because the data were clustered. A number of applications are received for each advertised commissioning brief and, of those, only one can be funded. In addition, the characteristics of the applications received for the same commissioning brief were likely to be correlated and were therefore not independent. We took account of this clustering allowing for intra-group correlation using the vce(cluster) option in STATA (the usual requirement that the observations be independent was relaxed). We considered observations (applications) to be independent across groups (commissioning briefs) but not necessarily within groups (as applications within groups were all in response to the same commissioning brief and in competition with each other).

To identify the characteristics most strongly associated with the application being shortlisted/not shortlisted and funded/not funded we used a backward and forward stepwise elimination process and Akaike information criteria to decide which characteristics were kept in the final model (as recommended by Royston et al. [39]). Akaike information criterion is a measure of model fit that includes a penalty against large models and hence attempts to reduce overfitting. For a single predictor, the criterion equates to selection at 15.7% significance [39]. We also fitted a full model, and compared the results to the models obtained from backwards and forwards selection methods. No interactions between characteristics were considered for inclusion in the models.

## Results

The number of outline proposals for primary research submitted to the NIHR HTA programme between January 1^st^ 2006 and 31^st^ December 2009 was 347 (Figure 1). One was ineligible for funding under the regulations of the programme. Of the remainder, 47 were in calls for which none of the proposals were shortlisted and 3 were asked to resubmit outline proposals; we excluded these from the analysis. This left a total of 296, from which we extracted data. Overall, 129 out of the 296 applications were shortlisted (43.6%) and 60/296 were funded (20%). The data are summarised in Table 2.

**Table 2:** **Results from conditional logistic regression modelling**

		Shortlisting results					Shortlisting model results		Funding model results	
Application characteristic	Sub-groups	No (%) with characteristic (n = 257)	Application shortlisted? No (%)		Univariate unadjusted odds ratio associated with shortlisting (95% CI) ^4*^	Unadjusted ***P***value ^4^	Shortlisted odds ratio (95% CI)*		Funded odds ratio (95% CI)*	
			Yes (n = 90)	No (n = 167)			Full model ^3^	Forwards/backwards model selection ^2^	Full model ^3^	Forwards/backwards model selection ^2^
**Characteristics**										
Statistician		164 (63.8%)	73 (81.1)	91 (54.5)	3.76 (2.21, 6.37)*	<0.0001	2.31 (0.97, 5.50)	2.25 (1.08, 4.68)*	6.20 (1.73, 22.27)*	6.23 (1.60, 24.28)*
Named clinician		227 (88.3%)	85 (94.4)	142 (85.0)	2.17 (0.65, 7.31)	0.2099	2.48 (0.43, 14.21)		1.28 (0.11, 15.54)	
Multidisciplinary team		205 (79.8%)	88 (97.8)	117 (70.1)	19.94 (5.13, 77.50)*	<0.0001	17.28 (2.59, 115.32)*	13.25 (1.93, 91.05)*	5.87 (0.87, 39.85)	3.56 (0.76, 16.67)
Number of applicants 4 or more		237 (92.2)	89 (98.9)	148 (88.6)	11.17 (1.36, 91.59)*	0.0246	0.44 (0.01, 14.92)		0.08 (0.01, 0.83)*	0.08 (0.01, 0.71)*
Pilot/feasibility study completed	Yes	10 (3.9%)	5 (5.6)	5 (3.0)	4.11 (1.24, 13.62)*	0.0209	14.76 (1.28, 169.89)*	9.07 (1.34, 61.49)*	16,971.42 (240.23, 1,198,987.00)*	2,563.64 (27.65, 237,671.6)*
	No^1^	44 (17.1%)	16 (17.8)	28 (16.8)	1.0		1.0		1.0	
	Unclear^1^	203 (79.0%)	69 (76.7)	134 (80.2)	1.0		1.0		1.0	
PI/team participated in systematic review		22 (8.6%)	11 (12.2)	11 (6.6)	1.63 (0.70, 3.78)	0.2590	0.37 (0.10, 1.35)		0.21 (0.05, 0.81)*	0.29 (0.09, 1.00)
PI gender	Male	165 (64.2%)	58 (64.4)	107 (64.1)	0.73 (0.39, 1.36)	0.3195	1.30 (0.54, 3.13)		0.47 (0.09, 2.40)	
PI Institution	Big 6	47 (18.3%)	16 (17.8)	31 (18.6)	1.10 (0.54, 2.22)	0.1223	1.32 (0.44, 3.90)		0.91 (0.27, 3.03)	0.93 (0.30, 2.89)
	Russell Gp exc. Big 6	103 (40.1%)	43 (47.8)	60 (35.9)	1.62 (1.00, 2.63)*		0.93 (0.41, 2.09)		0.04 (0.01, 0.26)*	0.08 (0.02, 0.39)*
	Other^1^	107 (41.6%)	31 (34.4)	76 (45.5)	1.0		1.0		1.0	1.0

*a star is shown next to an odds ratio and 95% confidence interval if it is significant at the 5% level (e.g., confidence interval excludes 1).

^1^Reference category (for characteristics for which possible categories were Yes, No, or Unclear the No and Unclear group were combined prior to inclusion in the regression models).

^2^Odds ratio and 95% confidence interval associated with the model coefficients of characteristics kept in the model following the selection process (forwards and backwards selection methods gave the same model so results for both have been presented in one column).

^3^Odds ratio and 95% confidence interval associated with the model coefficients from the model which included all characteristics.

^4^Odds ratio and 95% confidence interval with *P* value obtained by fitting a conditional logistic regression model on shortlisted/not shortlisted with just this characteristic included in the model.

Initial scoping, however, had not included the detailed examination of linked applications that were all rejected or funded and, therefore, non-contributory to the analysis. Because such linked events were non-contributory, only 53 of the 90 events contributed to the multivariate analyses on the outcome of being funded. We followed our pre-specified analysis plan for both shortlisted and funded and have presented the results of both in this publication.

### Univariate and multivariate statistical analysis

1. Analysis at shortlisting stage (Table 2)

Statistical analysis data for particular commissioning briefs where shortlisting outcomes were all positive were excluded (n = 39). Out of the 257 applications included in the analysis, 90 were shortlisted (35%). In the univariate analysis, the characteristics of the applicants positively associated with success at the first (shortlisting) board were having a named statistician, having a multidisciplinary team, larger teams with four or more applicants, and the completion of a pilot/feasibility study. There was no association with gender or institution of the first applicant, or with their participation in a systematic review. In the full model, multidisciplinarity of the team remained a significant association after adjustment, as did completion of a pilot or feasibility study. The association with larger teams did not remain after adjustment for other factors; however, this characteristic is related to multidisciplinarity. In the full model, neither the gender of the first applicant, nor the status of their institution was significantly linked to success at the board. The characteristics selected to be kept in the model from the forwards and backwards model selection process indicate the characteristics most strongly associated with success are presence of a named statistician on the application, multidisciplinarity of the team, and pilot/feasibility study completed.

We considered that previous research in a topic area by a formal systematic review would lead to better applications and be associated with success. We did not find a significant association with this but were limited by the small number of applications building on previous research. This should perhaps have been anticipated because the research questions were set by the funder, so most applicants were coming to the topic without the opportunity to undertake preliminary work.

2) Analysis at funding stage

Data for particular commissioning briefs where funding outcomes were either all positive or all negative for a commissioning brief (n = 21) were excluded from the statistical analysis because they contributed no useful data to the analysis; 60 out of the 236 applications included in the statistical analysis were funded. The success of the full application in obtaining funding at the second board was not the focus of this study. The funding decision is made on what is essentially a different application, which is submitted after input from the shortlisting board, potentially correcting deficiencies in the original application so these results should be interpreted with caution. The full model showed positive associations with having a named statistician, multidisciplinarity of the team, and the completion of a pilot or feasibility study. Larger teams with more than four applicants showed a negative association with funding. This result is unexpected and is possibly due to very low numbers of applications with small teams of applicants. Similarly, having participated in a systematic review and institution, being Russell Group excluding the big six, are also negatively associated with success. These results could also be due to low numbers in the model.

## Discussion

The main outcome of interest in this study was success at the first (shortlisting) stage where the outline applications are considered unmodified and without the board’s feedback. We examined features of the applications, determined *a priori* by our expert advisory group, that we anticipated might be associated with success or failure and a small number that we hoped would be unrelated to outcome. Of the latter, it was encouraging to find that the gender of the first applicant was not linked to success at either stage. This result was in agreement with the meta-analysis by Marsh et al., which investigated the effect of gender differences on peer review for grant applications, over a wide range of disciplines in different countries and found no difference [35]. Our result, like that of Marsh et al., was contrary to some previous findings, where being female may be disadvantageous in obtaining research funding [26–29, 32]. This difference may partly be accounted for by differences in the type of funding being sought, for example, the difference between personal fellowships and funding of projects undertaken by multidisciplinary teams. The status of the first applicants’ institution did not predict success either. We considered that applications from the most research intensive universities might be more successful; however, the fact that this was not so may reflect a wide distribution of good research skills and that many teams are collaborations between institutions. We were only able to easily retrieve the institution of the first applicant, and the teams are often made up from several institutions, diluting any effect from the first applicants’ institution. We did not find any evidence of favouring certain institutions, although over half of applications were from the Russell Group. Characteristics associated with success included those concerned with the multi-disciplinarity, particularly if the team included a statistician, and the completion of a pilot or feasibility study. The number of co-applicants is likely to be related to the multi-disciplinarity of the team [44]. In order for the complement of the team to include expertise in all relevant areas, proposals have to have a minimum number of collaborators which could explain why, in univariate analyses, applications with four or fewer applicants were less successful.

This study has several strengths; it was conducted using a data set from a large and established research funding programme, with continuity of process, covering a four year period. It benefited from the external advisory group who selected the characteristics in advance of the data extraction and analysis. Importantly, we were able to use a ‘commissioned research’ board to reduce the unknown confounding effect of the importance of the research question as a predictor of success since applicants were competing head to head to investigate the same question.

Interpretation is constrained by some limitations of the study. The study had only sufficient power to assess some of our parameters, even at the first assessment stage. We appropriately made the first stage of the application process our focus, where the outline form is being directly assessed, but limitation of the study was that we did not include an examination of the full applications. It would have been beyond the scope of this study to extract the data and incorporate these into the analysis. There may be a rather tenuous relationship between the initial application and the modified, often greatly improved, full proposal, and the lack of power due to only 60 studies being funded, means that we may have missed important predictors of success at the final funding board. This study examines one funding programme, using one dataset. The results have not been validated in a second dataset. We believe that the NIHR HTA Programme is similar to other applied research funders in that it uses a two stage application process assessed by committees and external peer review, although we acknowledge that commissioned research is rare. We suggest that our results are generalizable to other funders of applied research, but we have not tested this. Useful future research could include more detailed work examining full applications, a similar study examining researcher led applications, and a comparison looking at other research funders.

## Conclusions

Unlike previous reports from other research programmes, there was no evidence of an applicant’s gender or the status of their institution influencing the success of their application. This may reflect a more positive effort on behalf of the NIHR to ensure equal opportunities in line with the principles of Athena Swan. The characteristics most strongly associated with success are related to the range of expertise in the team, and the completion of previous pilot or feasibility study, and funding applicants could usefully take this into account.

### Study approval

This study did not require study ethics approval; it did not involve patients or clinical data.

## Abbreviations

CTU: Clinical Trials unit
HTA: Health Technology Assessment
NIHR: National Institute for Health Research.
